# Reducing RF-Induced Heating of DBS in 3 T MRI Using Dual-Role Receive Arrays as Wireless Resonators: A Simulation Study

**DOI:** 10.1109/access.2026.3713428

**Published:** 2026-07-14

**Authors:** ZHONGHAO ZHANG, MING LU, ZHENGYI LU, YUANKAI HUO, XINQIANG YAN

**Affiliations:** 1 Department of Electrical and Computer Engineering, Vanderbilt University, Nashville, TN 37235, USA; 2 Vanderbilt University Institute of Imaging Science, Vanderbilt University Medical Center, Nashville, TN 37232, USA; 3 Department of Computer Science, Vanderbilt University, Nashville, TN 37235, USA; 4 Department of Radiology and Radiological Sciences, Vanderbilt University Medical Center, Nashville, TN 37232, USA

**Keywords:** MRI safety, deep brain stimulation, RF-induced heating, E-field, wireless resonator, SAR optimization

## Abstract

Magnetic resonance imaging (MRI) scanning remains largely restricted to specific modalities, typically involving low radiofrequency (RF) power levels and stringent protocols for patients with deep brain stimulation (DBS) implants, due to safety concerns related to RF-induced heating of the implants. A 6-channel dual-role head coil array capable of modulating the electric-field (E-field) distribution was designed and evaluated using electromagnetic (EM) simulations. By optimizing the resonant frequency of each coil element during RF transmission, the transmit field was reshaped, leading to a significant reduction in RF-induced heating near the DBS lead tip. The proposed method was validated across two scenarios of increasing complexity: 1) a simple straight conductive wire for concept validation and 2) four realistic DBS leads representing complex real-world scenarios. The coil settings can be optimized either to suppress the E-field at a specific location, such as the DBS lead tip, or to suppress the peak specific absorption rate (SAR) across the entire human head. For location-specific E-field suppression, the simplified predefined-state control scheme and the fine-tuning genetic algorithm (GA)-based framework were implemented, achieving E-field reductions of 45.9% and 68.3%, respectively. For whole-head peak SAR suppression, the annealed Log-Sum-Exp (LSE)–Adaptive Moment Estimation (Adam) framework (LSE–Adam) was implemented, achieving an average 1 g SAR reduction of 72.09% across four realistic DBS lead models. The dual-role coil demonstrated a high degree of flexibility in controlling the transmit field and reducing RF-induced heating at DBS implants, offering a novel approach to mitigate RF-induced heating of the implants in MRI.

## I. INTRODUCTION

### A. MOTIVATION AND BACKGROUND

Deep brain stimulation (DBS) involves implanting electrodes in targeted brain regions to deliver electrical pulses, modulating neural activity. Since its inception several decades ago, the clinical utility of DBS has expanded substantially, with rapidly growing applications. It is a well-established therapy for conditions such as Parkinson’s disease, essential tremor, dystonia, and obsessive-compulsive disorder [1], [2], [3], [4], [5]. DBS delivers continuous electrical stimulation to targeted neural structures through chronically implanted electrodes connected to an internalized, programmable neurostimulator. The stimulation parameters, including amplitude, pulse width, and frequency, can be adjusted to meet individual therapeutic needs.

Because of its excellent soft-tissue contrast and high spatial resolution, magnetic resonance imaging (MRI) is a powerful non-invasive tool for addressing key questions in DBS. In clinical practice, DBS electrodes are implanted using stereotactic techniques, often guided by MRI to accurately localize the target brain regions, and are subsequently connected to a chronically implanted stimulator [6], [7]. However, radiofrequency (RF) heating induced by the transmit field remains a major safety concern for DBS during MRI. During RF transmission, the incident electric field (E-field) can induce currents along implanted leads [8], [9]. These currents propagate along the conductor and concentrate RF energy near the electrode tips, producing localized amplification of the E-field and specific absorption rate (SAR), which can cause substantial heating and potential tissue damage.

The RF-induced heating of DBS has been widely reported and can result in highly localized temperature increases near the electrode-tissue interface [8], [10]. This effect becomes stronger when the lead length is close to a resonant fraction of the RF wavelength in tissue. Its magnitude depends on the lead geometry, routing, and imaging conditions [11], [12].

### B. RELATED WORK

Currently, MRI exams for patients with DBS implants are performed under strict RF power limitations to prevent heating. Many patients with implanted devices are restricted to low-SAR protocols or lower field strengths [13], [14].

A widely adopted strategy for mitigating RF-induced heating is to modify the geometric trajectory of implanted leads. Baker et al. demonstrated that introducing concentric loops near the burr hole can significantly reduce RF-induced heating in DBS leads [15]. Building on this idea, Golestanirad et al. investigated the underlying mechanism using patient-specific computational models [16]. They showed that altering the lead trajectory changes the spatial distribution of the tangential component of the RF E-field along the conductor. Geometric trajectory modification reduces the effective induced voltage and current on the lead, thereby suppressing local SAR amplification and RF heating near the electrode tip.

Another strategy focuses on redesigning the DBS lead itself. Rather than modifying the implantation trajectory, these approaches alter the electromagnetic (EM) properties of the lead through resistively tapered or non-uniform conductor designs [17], [18].

In addition, extra conductive wires, named decoys, can be placed alongside the lead to reduce RF-induced heating [19]. These decoys couple with the main lead and help reduce the RF current induced on it. However, the applicability of all these approaches may be influenced by surgical constraints, hardware design considerations, and patient-specific lead configurations.

Meanwhile, Eryaman et al. demonstrated that modifying the transmit coil excitation to create regions of low or zero E-field can significantly reduce RF-induced heating of conductive implants [20], [21]. Subsequently, McElcheran et al. used parallel transmission (pTx), leveraging multiple independent RF channels to suppress the E-field near the implant while maintaining acceptable $B_{1}^{+}$ field homogeneity [22]. However, these approaches rely on aligning low-E-field regions with the implant and have only been validated for simple wire geometries. Golestanirad et al. proposed a mechanically rotatable linearly polarized birdcage transmit coil [23], [24]. A linearly polarized birdcage coil inherently exhibits slab-like regions of reduced E-field. By mechanically rotating the transmit coil, this low-E-field region can be steered to coincide with the implant trajectory, thereby minimizing the tangential E-field integrated along the lead and reducing the induced currents at the electrode contacts [23].

Subsequently, Kazemivalipour and Golestanirad et al. extended this approach to 3 T MRI and demonstrated its effectiveness in reducing RF-induced heating in bilateral DBS lead configurations [25], [26]. Kutscha and Keil et al. further advanced the approach by outfitting a 32-channel receive array at 3 T. This development brought the approach closer to clinical implementation [27].

Nevertheless, Bhusal et al. further evaluated lead management and reconfigurable coil approaches under more realistic anatomical conditions [28], [29].

Their results showed that the performance of these methods is strongly influenced by patient-specific factors. In addition, for reconfigurable coils, the performance is also sensitive to the operating conditions. For example, the worst-case MaxSAR of a linearly polarized birdcage coil can be up to 85% higher than that of a conventional circularly polarized birdcage [23]. This high sensitivity makes patient-specific EM simulations, which can take 16-32 hours per case [27] and impose a heavy burden on the clinical workflow. In addition, the mechanical complexity of the rotating coil system further limits its practicality.

More recently, pTx and RF shimming techniques have been explored as a means of reducing RF-induced heating of DBS implants. By independently controlling the amplitude and phase of multiple transmit channels, these methods can modify the E-field around implanted conductors. Guerin et al. demonstrated through simulations that 8-channel and 16-channel pTx coils can reduce absorbed power near DBS electrodes by more than 18-fold and 169-fold, respectively [30]. Zeng et al. and Chen et al. systematically evaluated the impact of RF shimming on implant heating at 3 T, showing that shimming conditions can significantly alter the RF-induced heating of implanted leads [31]. Recent work by Speybroeck et al. further demonstrated that even standard two-channel RF shimming on clinical 3 T scanners can meaningfully reduce heating near bilateral DBS leads [32]. While these pTx approaches show great promise, they typically require multi-channel transmit hardware and patient-specific calibration, which may limit their clinical deployment.

Wireless resonators, also known as inductively coupled resonators or wireless coils, have been widely used in MRI to manipulate RF fields [33], [34], [35]. Unlike active pTx systems, these resonators are not directly driven by RF power sources but instead interact with the primary transmit field through EM coupling. By appropriately tuning their self-resonant frequency, wireless resonators can generate secondary EM fields that are in phase or out of phase with the primary RF field, thereby reshaping both the E-field and transmit $B_{1}^{+}$ field distributions. Previous studies have used wireless resonators to enhance peripheral transmit fields at ultrahigh field strengths [33], [34]. Beyond transmit field manipulation, wireless resonators have been widely used to enhance the receive field $B_{1}^{-}$, thereby improving the signal-to-noise ratio (SNR) [30], [31], [32], [35], [36], [37], [38], [39], [40].

### C. OBJECTIVES AND CONTRIBUTIONS

In this work, we propose an alternative solution based on a single-layer coil whose behavior is controlled through the electrical circuitry. The coil array serves a dual role. During signal reception, the array operates as a conventional receive coil. During RF transmission, the same coil elements are frequency-shifted and function as wireless resonators, thereby enabling manipulation of the EM fields, while also reducing SAR [34], [41], [42], [43]. The proposed method was validated across two scenarios of increasing complexity: (1) a simple straight conductive wire and (2) four realistic DBS lead models. The coil settings can be optimized toward two objectives: location-specific E-field suppression, such as at the DBS lead tip, and whole-head peak SAR reduction. In addition, by leveraging co-simulation work [44], [45], we significantly reduced the simulation time for wireless resonators. Unlike approaches that rely on additional local transmit coils or mechanical coil-rotation systems, this method does not require extra hardware for RF heating reduction.

Building on this idea, our work extends the single-layer transceiver concept of Zhu et al. [41] to the DBS safety application. In the original design, each array element inductively couples with the body coil during transmission to enhance the local $B_{1}^{+}$ field and functions as a conventional receive element during reception. We retain this single-layer, dual-role architecture but make each element electronically tunable during transmission. This allows the elements to act as controllable wireless resonators that reshape the local *E*-field distribution, enabling *E*-field suppression near DBS leads and reducing RF-induced heating while preserving receive functionality.

## II. METHODS

### A. MODEL CONSTRUCTION

**FIGURE 1.**
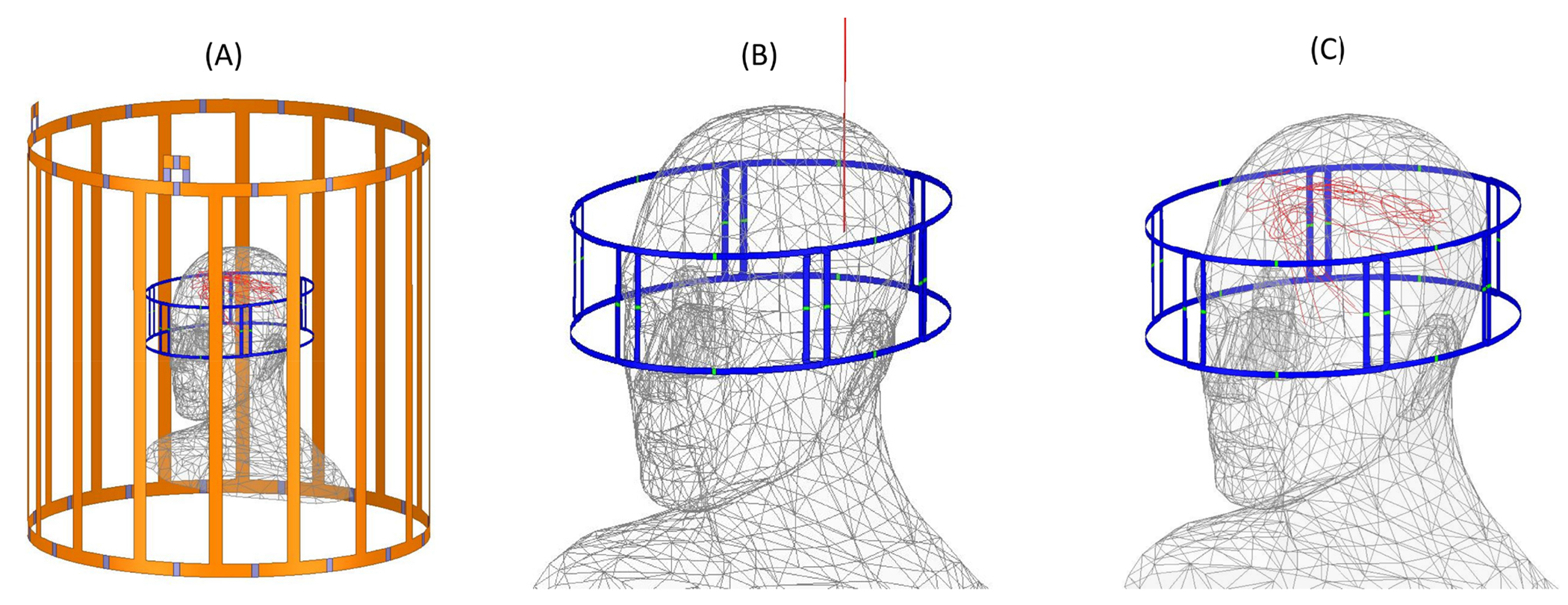
[A] Simulation model of a 3 T body transmit (Tx) coil combined with a decoupled six-channel wireless head coil array (blue). [B] Zoomed-in view of the 6-channel wireless resonator array with the birdcage coil model hidden. The image combines the human head and the simplified DBS lead model. The wireless resonator conductors are shown in blue, and the DBS electrode is shown in red. [C] Combined human head and DBS lead model. All DBS lead positions are shown together for illustration purposes; however, in the simulations, each DBS configuration was evaluated independently.

To validate the proposed method, two types of DBS models were evaluated in ANSYS HFSS(ANSYS Inc., Canonsburg, PA, USA) at 3 T. The first model was a simple straight conductive wire, which served as a proof-of-concept case. The second model consisted of four realistic DBS lead geometries, which were used to evaluate the performance under practical conditions. Both DBS models were embedded in a human head model provided by the vendor. The body transmit coil was modeled as a 16-rung high-pass birdcage coil with a diameter of 60 cm and a length of 60 cm, as shown in Fig. 1[A]. The conductors of the birdcage coil were modeled as copper with a finite conductivity of 5.8 × 10^7^ S/m and a width of 2 cm. The birdcage coil had two ports, tuned to 128 MHz and matched to 50 Ω, with isolation greater than 15 dB between ports (in the absence of passive resonators) across different loading conditions.

For both DBS configurations, a six-channel wireless resonator array was constructed inside the 3 T body coil. Each wireless resonator measured approximately 17 cm × 7 cm. To improve control of the head RF field, the coils were configured in an elliptical layout with dimensions of 28 cm × 22 cm. The conductors of the wireless resonators were modeled as copper with a finite conductivity of 5.8 × 10^7^ S/m and a width of 0.5 cm. Each wireless resonator element contained four capacitors, which are shown in green in Figure 1[B][C]. To simplify the tuning and control strategy, three capacitors were fixed at 25 pF for each wireless resonator, while only the top capacitor was kept tunable to adjust the resonant frequency of each coil.

Geometric overlap between adjacent wireless resonators was optimized to minimize mutual coupling and ensure independent operation of each element. The decoupling condition was verified using *S*_21_ measurements between adjacent coils. A single dominant resonance peak in the *S*_21_ response indicated minimized coupling between coil elements. This decoupling strategy was applied across the entire array to ensure stable and independent operation of all wireless resonators.

#### 1) STRAIGHT CONDUCTIVE WIRE MODEL

For the straight-wire DBS model (Fig. 1[B]), the lead had a diameter of 1 mm and a total length of 15 cm, with 6.5 cm embedded in the posterior region of a human head model provided by the vendor.

#### 2) REAL DBS MODELS

Fig. 1[C] illustrates four different DBS lead configurations. Each DBS lead represents a distinct implantation location corresponding to stimulation of a different brain region. The DBS leads were modeled as curved copper wires with a diameter of 1 mm and a finite conductivity of 5.8 × 10^7^ S/m. Although all DBS lead positions are shown together in Fig. 1[C] for visualization purposes, each configuration was simulated and optimized independently.

### B. PREDEFINED COIL-STATE OPTIMIZATION

**FIGURE 2.**
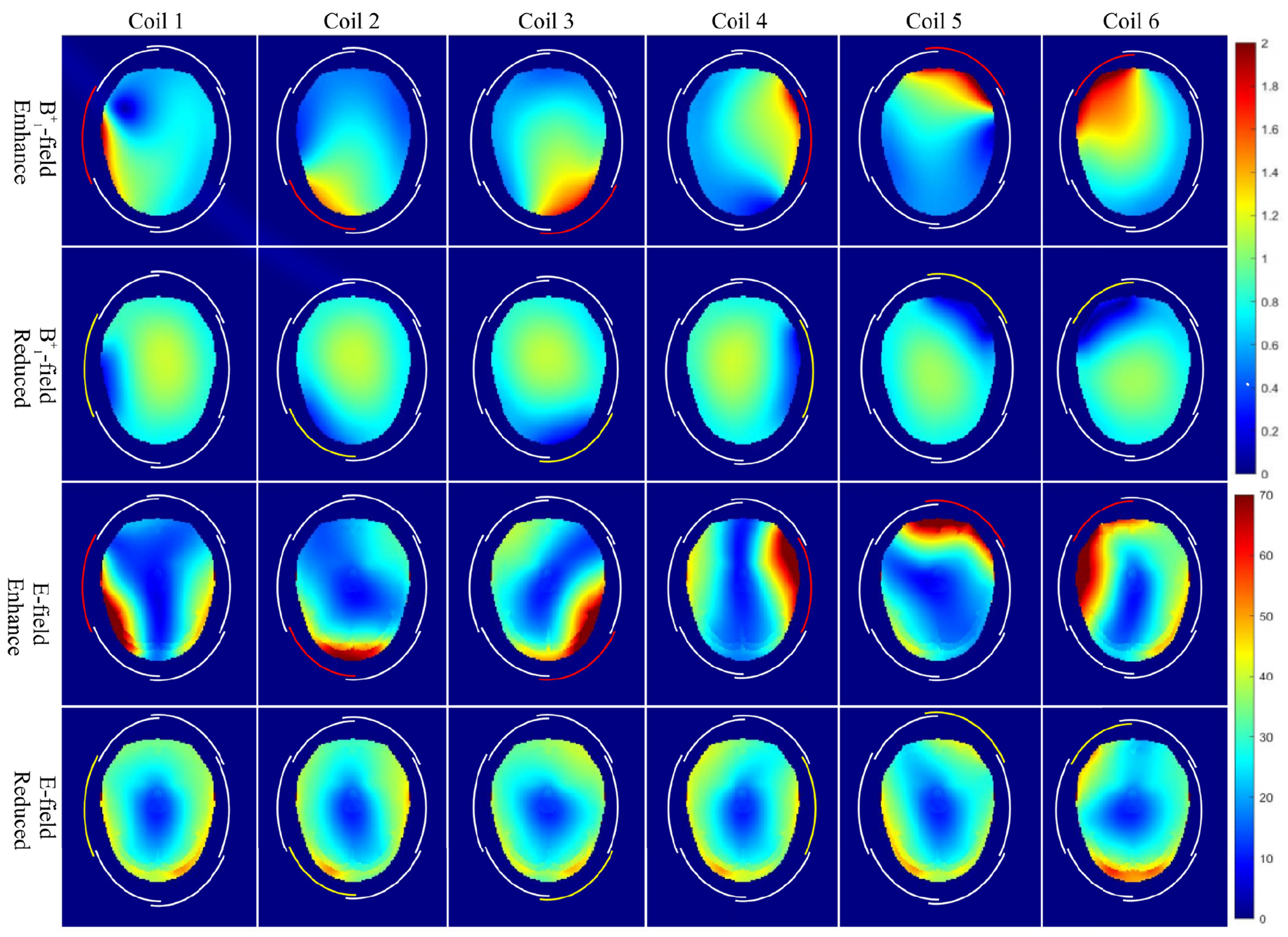
The $B_{1}^{+}$ and E-field distributions when each coil is activated individually. Each coil is assigned two predefined states: an enhanced state that increases the local RF field and a reduced state that decreases it. In the figure, white coils indicate detuned elements, red coils represent the enhanced mode, and yellow coils represent the reduced mode. Each coil element can be controlled independently, and the local RF field distribution is adjusted by switching the operating mode of each coil.

A simplified predefined control strategy was investigated to enable practical and robust operation. As shown in Fig. 2, in this scheme, each coil element in the six-channel dual-role array operated in one of three predefined states: enhanced, reduced, or detuned. These states correspond to fixed resonant frequency offsets that respectively increase, suppress, or eliminate the induced secondary EM fields generated by the wireless resonator. In practical scenarios, the state of each coil was adjusted according to the DBS lead location.

The predefined coil states were determined using EM co-simulations on a head model without a DBS lead. During the impedance-determination process, the impedance of one coil was swept to generate the corresponding transmit *B*-field distributions, while all remaining coils were disabled. From these generated field maps, the values corresponding to the maximum positive and negative impacts on the field in the head were selected manually as the enhanced and reduced states, respectively.

This procedure was repeated for all six coil elements, resulting in two predefined capacitor values for each coil. A detuned state was implemented by detuning the coil away from the Larmor frequency to minimize its coupling to the transmit field. Figure 2 illustrates the different operating modes of each coil element, where red indicates the enhanced mode, white indicates the detuned mode, and yellow indicates the reduced mode. Once established, these predefined states were reused across all DBS lead positions without further adjustment.

### C. FINE-TUNING EM-FIELD OPTIMIZATION

A precise tuning of the resonant frequency was performed based on a genetic algorithm (GA) to achieve improved control over the E-field and B-field. The proposed formulation incorporated both $B_{1}^{+}$ homogeneity and E-field suppression for excitation performance and safety. Therefore, the loss function jointly regulated both transmit-field uniformity and E-field exposure.

The loss function used to compute the reference impedance is given by

(1) $$ℒ(b)=\frac{std\left(B_{1}^{+}(b)\right)}{mean\left(B_{1}^{+}(b)\right)}+\gamma \frac{mean\left(E_{\text{target}}(b)\right)}{mean\left(B_{1}^{+}(b)\right)},$$

where *γ* is a weighting factor, $B_{1}^{+}(b)$ is the resulting transmit field produced by coil weights *b*, and $E_{\text{target}}(b)$ is the E-field magnitude evaluated within a selected target region. In this work, γ was set to 0.0196 based on empirical tuning. In this equation, both terms are divided by $B_{1}^{+}$ which served as a normalization factor for both terms to maintain a consistent excitation level during optimization.

The E-field penalty was applied only to a predefined target region, typically defined around the DBS lead trajectory:

(2) $$E_{\text{target}}=E⊙M_{\text{target}},$$

where $M_{\text{target}}$ was defined as a small 3D region of voxels surrounding the DBS lead tip. Because the lead-tip position varied across different DBS configurations, this target region was independently defined for each configuration.

The optimization used a GA-based search to optimize the resonant frequency of each coil by adjusting impedance variables, $\left[C_{1},C_{2},C_{3},C_{4},C_{5},C_{6}\right]$, within the bounds of 0.001–25 pF. At each iteration, new $B_{1}^{+}$ field was computed via co-simulation, and the loss function (Eq. 1) was evaluated to determine the performance score for that specific six-coil impedance combination.

### D. FINE-TUNING SAR OPTIMIZATION

Building on the Fine-Tuning EM-field Optimization, we further formulated a SAR-driven fine-tuning framework. The optimization objective of the proposed framework was to minimize the peak SAR over the entire head region while maintaining a consistent excitation level during optimization.

The loss function used to compute the reference impedance is given by

(3) $$ℒ(b)={ℒ}_{SAR}(b)+\gamma \frac{std\left(\left|B_{1}^{+}(b)\right|\right)}{mean\left(\left|B_{1}^{+}(b)\right|\right)}$$

where $B_{1}^{+}(b)$ is the resulting transmit field, ${ℒ}_{\text{SAR}}$ is discussed further in Eq. (4), and γ is a weighting factor. The std/mean form normalizes the $B_{1}^{+}$ penalty and prevents the optimization from collapsing into a trivial solution that simply suppresses the overall $B_{1}^{+}$ magnitude.

Because reducing SAR in one local region may increase SAR in another region during the optimization process, directly minimizing the SAR at a fixed location is not sufficient. A smooth maximum SAR objective was used to account for all voxel-wise SAR values within the whole-head mask. The smooth SAR objective is defined as

(4) $${ℒ}_{\text{SAR}}=\frac{1}{\alpha }\text{log}\left(\frac{1}{N}\sum_{i=1}^{N}\text{exp}\left(\alpha SAR_{\text{voxels}}\right)\right)$$

where ${\mathrm{SAR}}_{\text{voxels}}$ is the voxel-wise local SAR evaluated over all valid voxels within the whole human head region, *N* is the number of valid voxels, and α is applied inside the exponential function to increase the contribution of high-SAR voxels. As α increases, the loss function pays more attention to voxels with higher SAR values.

At each iteration, the E-field distribution and voxel-wise SAR were recalculated, and the impedances were updated using Adaptive Moment Estimation (Adam)-based optimization. After convergence, the peak 1 g-averaged SAR was computed at the optimized impedance configuration (*C*_1_–*C*_6_) using the standard spherical averaging procedure. To ensure that each capacitor value stayed within the available tuning range, an intermediate variable *u_i_* was used, and it was converted to the physical capacitor value using

$$C_{i}=C_{min}+\left(C_{max}-C_{min}\right)\frac{1}{1+e^{-u_{i}}}.$$

The sigmoid function maps any value of $u_{i}$ into the range (0, 1), which constrains the resulting capacitor value $C_{i}$ between $C_{\text{min}}$ and $C_{\text{max}}$. The Adam optimizer [46] was applied to the unconstrained variable $u_{i}$.

## III. RESULTS

### A. PREDEFINED COIL-STATE OPTIMIZATION

In this approach, each wireless resonator element was restricted to three predefined operating modes: enhanced, reduced, and detuned. For each coil element, one coil was activated while all remaining coils were detuned, and the capacitor value of the active coil was swept to evaluate its effect on the local RF field. Two capacitor settings corresponding to the maximum enhancement and suppression of the local RF field were selected and defined as the enhanced and reduced modes, respectively, while the detuned mode was implemented by shifting the coil away from the Larmor frequency to minimize coupling.

**FIGURE 3.**
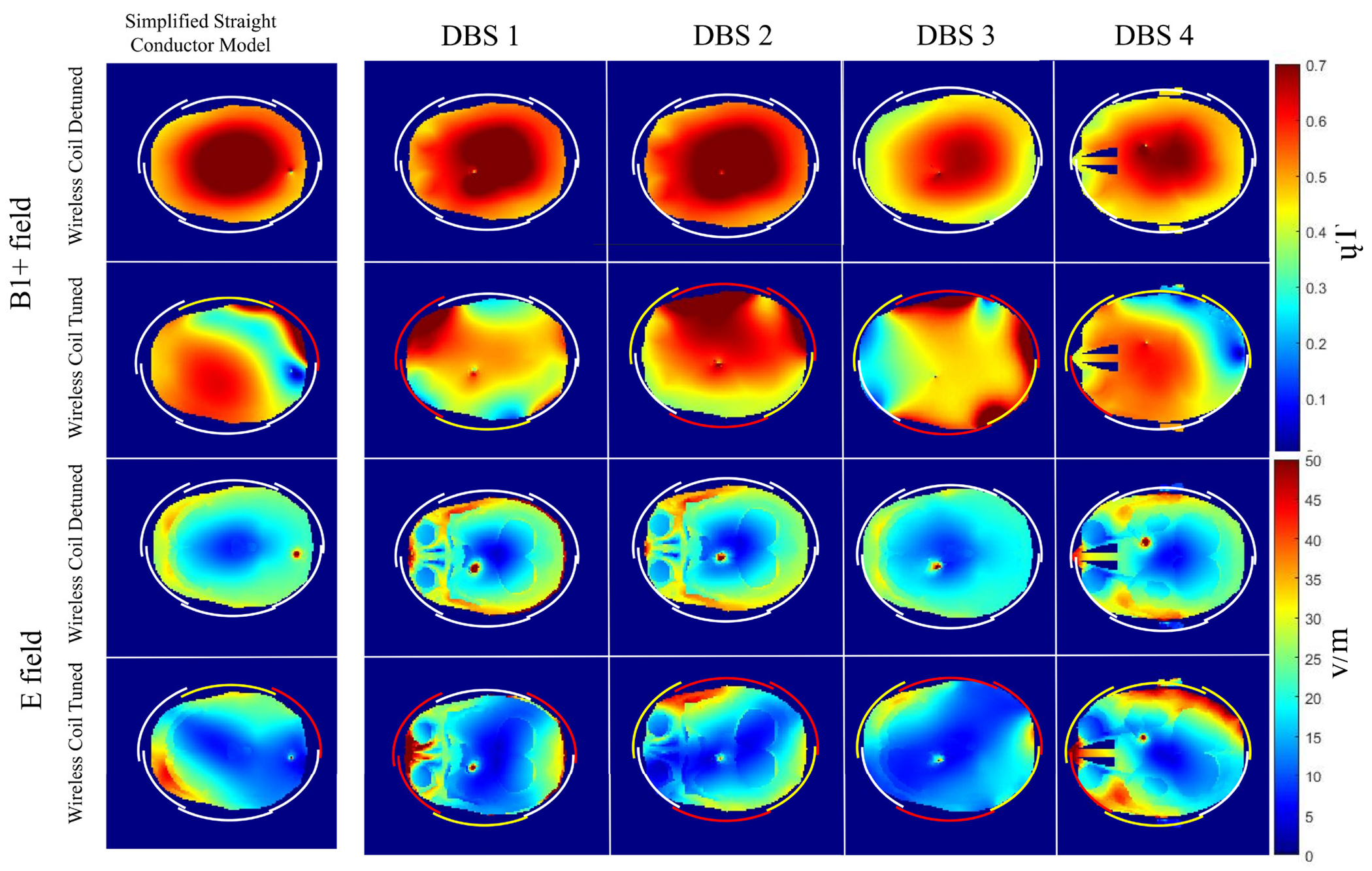
Comparison of the DBS lead configurations before and after wireless resonator tuning. The displayed slice is taken at the tip of the DBS lead, where RF-induced heating is most severe. Unlike Fig. 4, the capacitor values used in this case are predefined, and each wireless resonator operates in one of three discrete modes: enhanced, reduced, or detuned. The wireless resonator tuning still substantially reduces the E-field near the DBS lead tip.

The coil impedance in this predefined strategy could not be continuously adjusted and instead had to be selected from the predefined states. This constraint significantly reduced the optimization complexity and computational cost. Figure 3 presents the results for five different DBS lead configurations using this predefined control scheme, where the operating states of all wireless resonators were determined manually. Table 1 presents the quantitative details of the E-field reduction and the transmit $B_{1}^{+}$ field uniformity using predefined wireless resonator states.

### B. FINE-TUNING EM-FIELD OPTIMIZATION

**FIGURE 4.**
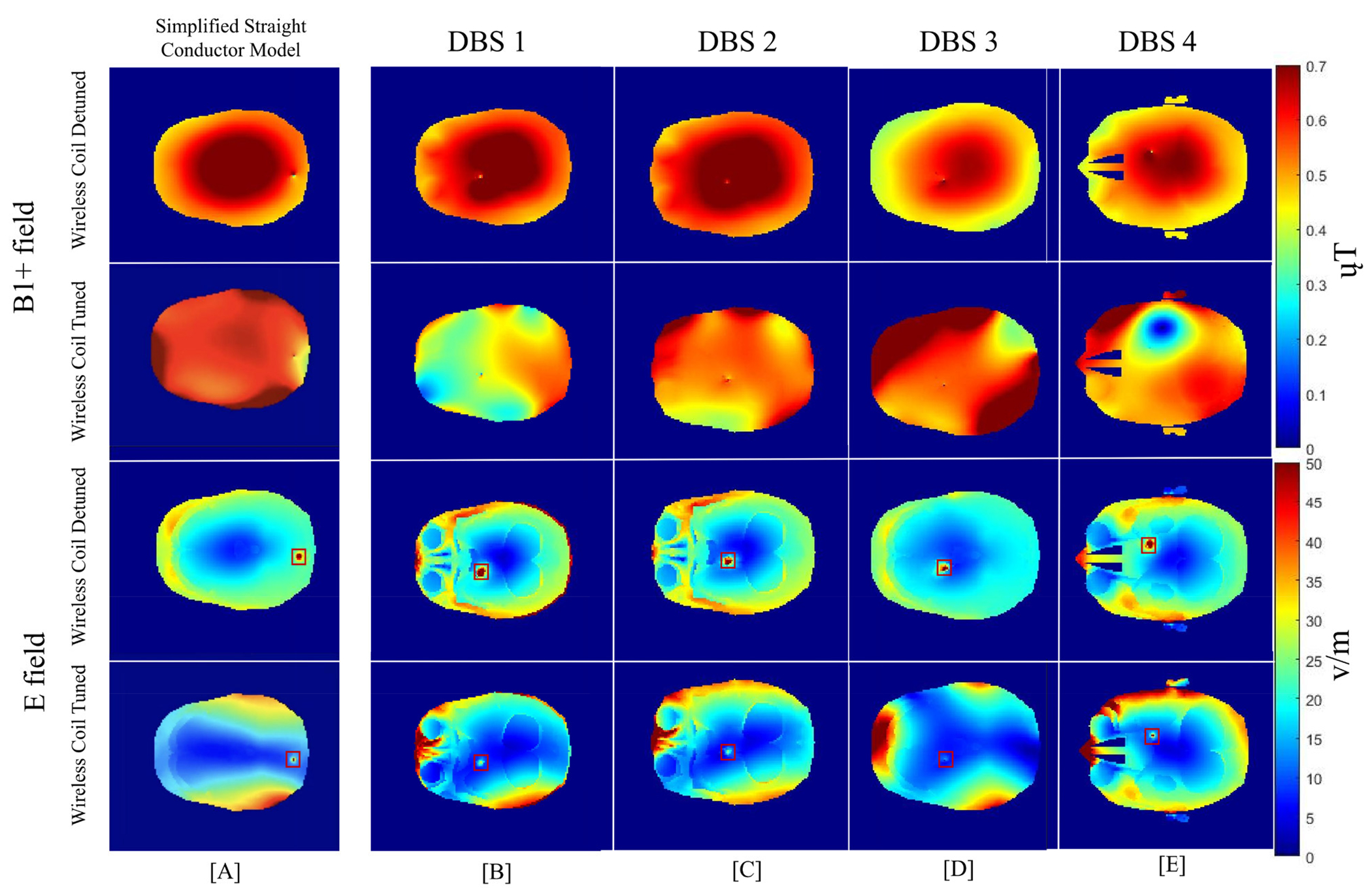
The figure compares five different DBS lead configurations before and after wireless resonator tuning. [A] corresponds to an idealized straight DBS lead, whereas [B]-[E] represent realistic DBS lead geometries. The displayed slice is taken at the tip of the DBS lead, where RF-induced heating is most severe. The objective of the wireless resonator tuning is to minimize the E-field in the region near the DBS lead tip, as indicated by the red boxed area. During optimization, the capacitor values are allowed to vary continuously within the range of 0.001 to 25 pF. After tuning, the wireless resonator substantially reduces the E-field at the DBS tip while preserving the overall uniformity of the transmit $B_{1}^{+}$ field.

A GA optimization framework was employed to tune the six capacitors (*C*_1_–*C*_6_) of the wireless resonator array, with each capacitor constrained to the range of 0.001–25 pF. The termination criterion was set to a function tolerance of 10^−4^. A population size of 80 individuals was used, with the crossover fraction (0.8) and adaptive feasible mutation function from the MATLAB Toolbox. A maximum of 100 generations was further imposed as a safeguard. The optimization objective was to reduce the E-field magnitude within a localized region near the DBS lead tip (highlighted by the red box in Fig. 4).

Figure 4 compares the E-field and $B_{1}^{+}$ field distributions with and without wireless resonator modulation. When all wireless resonator elements were fully detuned, the resulting fields resembled those of a conventional body coil, exhibiting an elevated peripheral E-field and a spatially uniform $B_{1}^{+}$ field. A pronounced increase in the E-field was observed near the electrode tip. With wireless resonator modulation, the E-field in the DBS vicinity (highlighted by the red box) was substantially reduced, particularly at the lead tip where RF-induced heating is most severe, while the overall $B_{1}^{+}$ field uniformity was well preserved. For each DBS configuration, the optimization required approximately 30 minutes. Table 2 presents the quantitative details of the E-field reduction and the transmit $B_{1}^{+}$ field uniformity.

### C. FINE-TUNING SAR OPTIMIZATION

The annealed Log-Sum-Exp (LSE)–Adam framework was used to tune the six capacitor values *C*_1_–*C*_6_ of the dual-role coil array. Each capacitor was limited to the range [0.001, 25] pF. The main objective of this optimization was to reduce the peak SAR over the whole head region. The framework used a differentiable forward model that mapped the voxel-wise SAR distribution to the capacitor vector. Because the loss function was non-convex, the optimization was repeated from 100 different random starting points to reduce the chance of being trapped in a local minimum. For each restart, Adam was run for 10,000 iterations. After all restarts, the candidate capacitor vector that achieved the lowest peak 1 g-averaged SAR was selected as the final solution. Because the EM-field modulation capability of the dual-role receive array had already been validated using the previous two methods, the straight-wire model was not further used in this analysis.

Table 3 summarizes the 1 g-averaged SAR reduction obtained after SAR-based fine-tuning for each lead configuration. Unlike the previous two figures, both the $B_{1}^{+}$ std/mean and the 1 g-averaged SAR values here are evaluated over the entire head volume. Because the absolute 1 g-averaged SAR values depend on excitation power and the location of the maximum 1 g-averaged SAR may change with coil tuning, the results are reported as percentage reductions in peak 1 g-averaged SAR and the $B_{1}^{+}$ std/mean over the whole-head region.

The annealed LSE–Adam framework reduced the peak SAR over the whole head region while maintaining the $B_{1}^{+}$ field uniformity, as reflected by the $B_{1}^{+}$ std/mean values. The optimization required approximately 12 hours for each DBS configuration on an NVIDIA Quadro P5000 GPU.

## IV. DISCUSSION

### A. SUMMARY

We presented a framework for mitigating RF-induced heating and SAR associated with DBS implants during MRI. A dual-role receive array was leveraged to operate as a wireless resonator during the transmit phase. By tuning the resonant frequencies of the array elements, the proposed method enabled effective manipulation of the *E*-field and reduction of peak SAR.

The framework was validated using two DBS models of increasing complexity: an idealized straight conductive wire, and multiple realistic DBS lead trajectories embedded in a human head model. Based on these models, three optimization strategies were developed to target two objectives: location-specific *E*-field suppression and whole-head peak SAR reduction. For location-specific *E*-field suppression, we implemented a simplified predefined-state control scheme and a fine-tuning GA-based framework, both tailored to reduce the *E*-field around the DBS lead-tip location. For whole-head peak SAR reduction, we developed an annealed LSE–Adam SAR optimization method that continuously adjusted the tuning capacitor values to reduce peak SAR.

Simulation results in ANSYS HFSS at 3 T showed that the proposed system effectively reduced the *E*-field near the DBS implants and suppressed SAR in the human head. The predefined-state method and the GA-based framework achieved reductions of 45.9% and 68.3%, respectively, in the average *E*-field magnitude near the DBS lead-tip region. The annealed LSE–Adam SAR optimization achieved an average 72.09% reduction in peak 1 g-averaged SAR over the entire head volume. Overall, the proposed wireless resonator modulation effectively reduced the local E-field near the DBS lead tip or the peak 1-g averaged SAR. Unlike approaches that rely on additional local transmit coils, mechanical coil-rotation systems, or redesigned DBS implants, this method does not require extra hardware for RF-heating reduction. Moreover, it demonstrates a high degree of flexibility in controlling the transmit field and mitigating RF-induced heating at DBS implants, offering a promising solution for safer MRI in DBS patients.

### B. COMPARISON OF THE THREE OPTIMIZATION STRATEGIES

The three optimization strategies provided different balances between SAR reduction, computational cost, and clinical practicality.

First, the predefined coil-state strategy was the simplest method. In this approach, each wireless resonator was limited to a small number of predefined capacitor states. This method did not require patient-specific optimization and was therefore the easiest to implement in practice. Across all DBS configurations, it reduced the average E-field magnitude near the DBS lead tip from 46.36 V/m to 24.50 V/m, corresponding to a 45.9% reduction. However, because only a few discrete coil states were available, the field control was limited and the improvement was smaller than those achieved by the other two methods.

Second, the GA-based Fine-Tuning EM-field optimization provided stronger E-field suppression by continuously tuning the coil resonant frequencies according to the DBS lead geometry. This method reduced the average E-field magnitude near the DBS lead tip from 46.36 V/m to 14.71 V/m, corresponding to a 68.3% reduction. It also included the $B_{1}^{+}$ std/mean term in the optimization objective, which helped preserve transmit-field uniformity. This method targeted the E-field magnitude in a localized region near the DBS lead tip, rather than directly optimizing SAR.

Third, the annealed LSE–Adam framework directly targeted SAR reduction rather than only reducing the local E-field near the lead tip. The optimization was performed on the peak voxel-wise local SAR because it provides an efficient and differentiable objective for gradient-based optimization. Although the peak 1 g-averaged SAR was not directly minimized during the optimization, reducing the peak voxel-wise local SAR suppresses the underlying |*E*|^2^ hotspots that contribute to elevated 1 g-averaged SAR. This method minimized the peak SAR over the whole head and achieved an average reduction of 72.09% in peak 1 g-averaged SAR. However, it had the highest computational cost, requiring approximately 12 hours per DBS configuration on an NVIDIA Quadro P5000 GPU.

### C. COMPARISON WITH EXISTING RF-HEATING MITIGATION TECHNIQUES

The performance of the proposed framework is evaluated through comparison with existing RF-heating mitigation strategies. Because these strategies were developed under different conditions, a direct numerical comparison is not feasible. This section provides a qualitative comparison in terms of RF-heating/SAR reduction, hardware complexity, computational burden, and clinical feasibility.

The most widely adopted strategies modify the implant itself. Surgical trajectory modification reduced the peak 1 g-averaged SAR at the contralateral lead tip by up to 18-fold at 3 T and by 80% at 1.5 T [15], [16]. Lead-redesign methods show similar levels of improvement. A resistively tapered conductor reduced simulated 0.1 g SAR by about 7 times [17]; a graded-conductivity design lowered the in-vitro tip temperature rise by more than 60% [18]; and coupled decoy conductors reduced measured tip heating by about 97% [19]. These implant-side methods do not need additional scanner-side hardware and per-scan optimization, but they require altered surgical practice or new implant designs. They cannot be applied retrospectively to already-implanted patients.

Reconfigurable rotating linearly polarized birdcage coils steer a slab-like low-E-field region onto the lead trajectory. According to the reduction performance reported in the study, in-vitro measurements at 1.5 T reported average heating reductions of 80% for unilateral and 65% for bilateral leads [26], while at 3 T the SAR was reduced on average by 83% (unilateral) and 59% (bilateral) [25]. This improvement increased to more than 90% when combined with surgical lead management. The 3 T prototype further achieved 76–92% SAR reduction and 92–97% measured heating reduction across realistic trajectories [27]. In terms of hardware complexity, however, this approach requires a mechanically rotating transmitter. It also requires a patient-specific EM simulation to predict the optimal rotation angle (approximately 16-32h per patient-specific model [27]). The clinical practicality is limited by the mechanical rotation mechanism and a strong sensitivity to the operating angle.

Parallel-transmit uses several RF sources to move the electric field away from the implant. McElcheran et al. reported 95–99% E-field suppression at the lead tip in simulation and an approximately 90% heating reduction with a four-channel prototype at 3 T [22]; Guerin et al. reported reductions of absorbed power near DBS electrodes exceeding 18-fold and 169-fold with 8- and 16-channel pTx coils, respectively [30]. The dual-drive birdcage reduced the tip temperature rise from more than 4.9 °C to less than 0.3 °C [21]. This performance requires multiple independent transmit channels and dedicated RF amplifiers. The computational burden includes patient-specific pulse or shim optimization. In terms of clinical practicality, this approach requires multi-channel transmit hardware and patient-specific calibration.

The proposed dual-role array achieves competitive RF-heating reduction with relatively low hardware complexity. It reduced the local E-field near the lead tip by 45.9% with the predefined scheme and 68.3% with the GA-based scheme, and achieved a 72.09% average reduction in peak 1 g-averaged SAR over the whole head. The method reuses the receive array as a tunable wireless-resonator system during transmission, avoiding extra transmit amplifiers, mechanical rotation, or DBS lead modification.

### D. EXTENSION TO OTHER APPLICATIONS

Although this study was conducted using a six-loop wireless resonator array, the proposed method is readily extendable to more complex geometries and larger multi-element arrays for improved transmit field control. For example, the method can be applied to a 32-element wireless resonator array, where the increased number of elements provides additional degrees of freedom, enabling more precise manipulation of the transmit field. Moreover, the proposed method is not restricted to brain applications and can potentially be extended to transmit field control in other anatomical regions, such as musculoskeletal imaging (e.g., the knee). The wireless resonators may help improve field uniformity and reduce localized RF exposure.

### E. LIMITATIONS OF THIS WORK

It should be noted that continuous tuning of capacitors in wireless resonator arrays remains challenging in practical implementations. Precise adjustment of lumped capacitors typically requires additional hardware complexity, calibration effort, and reliable control mechanisms, which may limit clinical feasibility.

To address this practical constraint, a simplified predefined coil-state strategy was introduced. In this strategy, each wireless resonator element operates in a small set of discrete states. However, this predefined approach restricts the available degrees of freedom in coil tuning and does not allow continuous optimization of the resonant frequency. As a result, compared with location-specific continuous tuning, the predefined strategy involves a trade-off in transmit field control performance.

In addition, a trade-off between SAR or E-field suppression and transmit-field uniformity was observed across all three optimization strategies. This trade-off arises because the wireless resonator elements modulate the transmit EM field as a whole; therefore, adjustments that reduce the local E-field or SAR can also alter the $B_{1}^{+}$ field distribution. As a result, the E-field/SAR reduction and $B_{1}^{+}$ homogeneity cannot be optimized completely independently. For example, in the annealed LSE–Adam SAR optimization, the $B_{1}^{+}$ std/mean increased from 0.2476 to 0.3066 in DBS 1 and from 0.2214 to 0.2528 in DBS 4, despite a substantial reduction in peak 1 g-averaged SAR. Depending on the clinical priority, the relative weighting between SAR/E-field suppression and $B_{1}^{+}$ homogeneity can be adjusted to balance these competing objectives.

Furthermore, the proposed framework was validated using a single anatomical head model and a limited number of DBS lead trajectories. Because RF-induced heating is highly sensitive to patient-specific anatomy, body composition, and implant routing [28], [29], different head models or lead trajectories may require a new, computationally intensive optimization process for each case, which was not evaluated in the present study. Future work should evaluate the robustness of the proposed framework across multiple anatomical models and a broader range of implant routings to better characterize its generalizability before clinical translation.

## V. CONCLUSION

In this work, we presented a dual-role receive-array framework for mitigating RF-induced heating associated with DBS implants during MRI. During signal reception, the array operated as a conventional receive coil, while during RF transmission, it functioned as a wireless resonator array. By tuning the resonant frequency of each coil element, the proposed method enabled the modulation of the transmit EM field without requiring additional transmit hardware, mechanical coil rotation, or modification of the DBS lead structure. EM simulations using both an idealized conductive-wire model and realistic DBS lead trajectories demonstrated that the proposed strategy could suppress local *E*-field hotspots near the DBS lead tip and reduce peak SAR within the human head.

Three optimization strategies were investigated for different levels of control complexity and safety objectives. The predefined-state control scheme reduced the average *E*-field magnitude near the DBS lead-tip region by 45.9%, while the GA-based location-specific EM-field optimization achieved a 68.3% reduction. For whole-head SAR mitigation, the annealed LSE–Adam optimization framework achieved an average 72.09% reduction in peak 1 g-averaged SAR while maintaining transmit-field homogeneity. These results demonstrate that the electronically tunable dual-role receive-array framework provides an effective and flexible approach for reducing DBS-related RF heating during MRI.

Overall, the proposed framework provides an efficient pathway toward improving MRI safety for patients with DBS implants. By integrating RF-heating mitigation into a dual-role receive-array architecture, this approach preserves receive functionality while enabling adaptive transmit-field control. Future work will focus on experimental validation, implementation in more complex geometries and larger multi-element arrays, and evaluation under additional patient-specific DBS lead configurations.

## Figures and Tables

**TABLE 1.** Results of the predefined wireless-resonator strategy for each individual lead configuration. For each configuration, the *E*-field row lists the spatially averaged |*E*| over the target region near the DBS lead tip, and the $B_{1}^{+}$ row lists the transmit-field inhomogeneity (std/mean).

Metric	Straight Conductor	DBS 1	DBS 2	DBS 3	DBS 4
*E* field (V/m)	44.98→15.27	49.75→29.54	35.67→26.04	41.22→23.60	60.20→28.05
$B_{1}^{+}$ std/mean	0.1591→0.26.39	0.1462→0.2368	O.1393→O.239I	0.1638→0.3264	0.1545→0.3066

**TABLE 2.** Results of the fine-tuning EM-field (GA-based) optimization for each individual lead configuration. For each configuration, the *E*-field row lists the spatially averaged |*E*| over the target region near the DBS lead tip, and the $B_{1}^{+}$ row lists the transmit-field inhomogeneity (std/mean).

Metric	Straight Conductor	DBS 1	DBS 2	DBS 3	DBS 4
*E* field (V/m)	44.98→11.50	49.75→15.28	35.67→12.85	41.22→12.59	60.20→21.33
$B_{1}^{+}$ std/mean	0.1591→0.1326	0.1462→0.2049	0.1393→0.1330	0.1638→0.3101	0.1545→0.2654

**TABLE 3.** Results of the fine-tuning SAR optimization for each realistic DBS configuration. The SAR row lists the reduction in peak 1 g SAR (%), and the $B_{1}^{+}$ row lists the inhomogeneity (std/mean) before→after tuning; both are evaluated over the entire head volume.

Metric	DBS 1	DBS 2	DBS 3	DBS 4
Peak 1 g-averaged SAR reduction (%)	47.91%	73.27%	88.82%	78.37%
$B_{1}^{+}$ std/mean	0.2476→0.3066	0.2361→0.2525	0.3080→0.2680	0.2214→0.2528
